# Dynamics of miRNA transcriptome during gonadal development of zebrafish

**DOI:** 10.1038/srep43850

**Published:** 2017-03-06

**Authors:** Christopher Presslauer, Teshome Tilahun Bizuayehu, Martina Kopp, Jorge M. O. Fernandes, Igor Babiak

**Affiliations:** 1 Faculty of Biosciences and Aquaculture, Nord University, 8049 Bodø, Norway

## Abstract

Studies in non-teleost vertebrates have found microRNAs (miRNAs) to be essential for proper gonadal development. However, comparatively little is known about their role during gonadal development in teleost fishes. So far in zebrafish, a model teleost, transcript profiling throughout gonadal development has not been established because of a tiny size of an organ in juvenile stages and its poor distinguishability from surrounding tissues. We performed small RNA sequencing on isolated gonads of *See-Thru-Gonad* line, from the undifferentiated state at 3 weeks post fertilization (wpf) to fully mature adults at 24 wpf. We identified 520 gonadal mature miRNAs; 111 of them had significant changes in abundance over time, while 50 miRNAs were either testis- or ovary-enriched significantly in at least one developmental stage. We characterized patterns of miRNA abundance over time including isomiR variants. We identified putative germline versus gonadal somatic miRNAs through differential small RNA sequencing of isolated gametes versus the whole gonads. This report is the most comprehensive analysis of the miRNA repertoire in zebrafish gonads during the sexual development to date and provides an important database from which functional studies can be performed.

MicroRNAs (miRNAs) are a highly conserved class of small regulatory RNAs, approximately 22 nucleotides (nt) long, which have a primary function in repressing the post-transcriptional expression of target messenger RNAs (mRNAs)^1^. The functions of miRNAs are diverse, including regulation of cell division^2^, differentiation^3^, migration^4^, and apoptosis^5^. However, most knowledge on miRNA in vertebrates is restricted to mammals. As of 2014, 11,717 mature miRNAs have been identified in mammals, compared to only 1,044 in teleost fishes^6^, despite teleosts being distinctively the most speciose group of all vertebrates with over 23,500 described species^7^.

The function of miRNAs during gonadal development is currently a subject of debate. In the more common canonical miRNA biogenesis pathway, miRNAs are transcribed as long primary transcripts (pri-miRNA)^8^. Within the nucleus, pri-miRNAs are recognized by the microprocessor Drosha/DGCR8 complex, which cleaves the miRNA hairpin to produce the precursor miRNA (pre-miRNA)^8^. After export to the cytoplasm, the pre-miRNA is processed by the enzyme Dicer to produce an RNA duplex, which is loaded into an Argonaute (Ago) protein^8^. In mouse (*Mus musculus*), germline mutations for either Dicer or Ago resulted in oocytes which were unable to complete meiosis and were sterile^9^,^10^,^11^. Similarly, ablation of *Dicer1* in gonadal somatic cells resulted in widespread suppression of miRNA expression, leading to abnormal gonad morphology and sterility^12^,^13^. By comparison, knockout of Dicer in male mice affected germ cell and spermatogonia proliferation and differentiation, but Ago2 deficient testis developed normally^14^. In another study on mouse, *Dgcr8* deficient oocytes were able to develop normally^15^. The authors found evidence that since Dicer also processes small interfering RNAs (siRNAs)^8^, it is likely their loss which explained the previous Dicer mutant phenotypes in oocytes, whereas miRNA activity is suppressed during oocyte maturation^15^. However, the authors also reported diminished brood size in *Dgcr8* knockout mice, suggesting a role for maternal miRNAs^15^.

Studies in zebrafish have found miRNAs to be essential for proper development. In *dicer1* mutants, developmental arrest was observed at approximately 10 days, after maternal Dicer1 activity has ceased^16^, while maternal dicer mutant embryos had severe morphological deformities early during embryogenesis^17^. However, the role of miRNAs during gonadal development is unclear. Zebrafish with germline *dicer* and *ago2* mutations are able to reach sexual maturity and produce offspring^17^,^18^. Instead, both Zili, and Ziwi, two proteins from the Piwi subclass of Argonaute proteins which interact with Piwi-interacting RNAs (piRNAs), are required for germline meiosis and survival^19^,^20^. The primary functions of teleost gonadal miRNAs may therefore be related to gonadal-somatic cell development or interactions between somatic and germ cells. Establishing miRNA repertoires between germline and gonadal-somatic cells may help to address this question.

In recent years, several studies have reported differential expression of miRNAs during gonadal development of commercially important teleost species. The first study was performed in Atlantic halibut (*Hippoglossus hippoglossus*), where sexually dimorphic miRNA expression was detected between immature testis and ovaries, as well as between sexually mature and immature testis^21^. Further studies in rainbow trout, (*Oncorhynchus mykiss*) have identified miRNAs in several developmental stages of testes, ovary, and unfertilized egg^22^,^23^. In yellow catfish (*Pylodictis fulvidraco*), the miRNA repertoire of immature gonads was determined^24^, and in Nile tilapia (*Oreochromis niloticus*), gonads during sexual differentiation^25^ as well as at sexual maturity have been sequenced^26^. While these studies provide important information about the miRNA repertoire during specific stages of development, the findings lack context without a more complete overview of the gonadal miRNA repertoire. As of yet, no study is reported to characterize miRNAs in both testis and ovary throughout gonadal development in a teleost. A more complete overview of miRNA abundance would be beneficial to understand the role of miRNAs in teleost reproduction.

Zebrafish (*Danio rerio*) is a model teleost species for developmental biology. However, due to its small size, sequencing of small transcriptome from zebrafish gonads has thus far been restricted to sexually mature organs^27^ and spermatozoa^28^ only. To overcome the challenge of distinguishing zebrafish gonads during juvenile stages of development, we have recently established *See-Thru-Gonad* line^29^; this line is a hybrid of the zf45Tg^30^ and *nacre transparent* (−/−)^31^,^32^ lines. In *See-Thru-Gonad* fish, the germline is visualized *in vivo* under epifluorescent light during its lifetime^29^.

The objective of the present study was to explore the miRNA repertoire during gonadal development of the zebrafish. We isolated gonads throughout gonadal development of *See-Thru-Gonad* line, from undifferentiated gonads at 3 weeks post fertilization (wpf) to fully mature gonads at 24 wpf. We also sampled mature oocytes and spermatozoa. With the exception of 3 wpf gonads and sperm, which were pooled for sequencing, we performed small RNA sequencing on individual testes and ovaries throughout gonadal development assuring true biological replications. For this purpose, we developed RNA low-input protocol for library construction and tested its reliability for scarce sample amounts. For the first time, we characterized miRNA dynamics during zebrafish gonadal development.

## Results

### Sequence statistics

**Figure 1:**
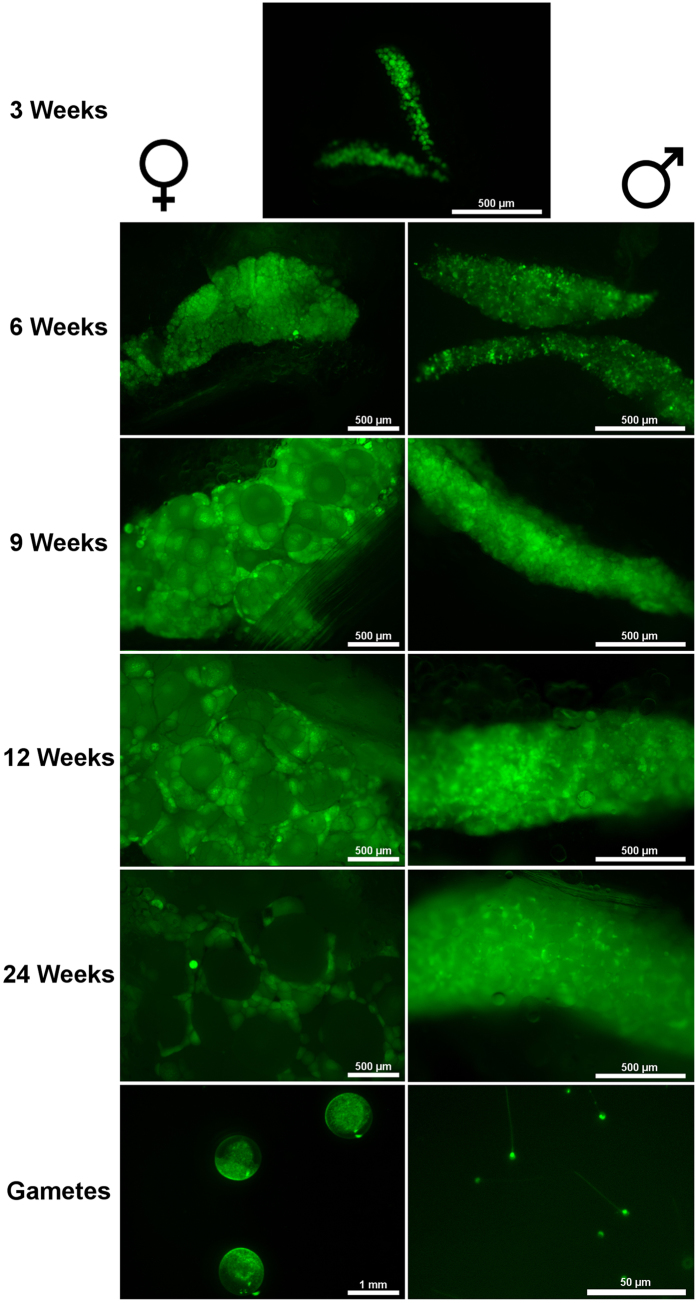
Representation of zebrafish gonads expressing vasa:vasa-eGFP transgene at each sequenced gonadal stage.

Zebrafish gonads from nine stages of development, as well as gametes (Fig. 1) were sampled and used to prepare a total of 47 small RNA libraries. Sequencing of these libraries generated over 227 million reads (Supplementary Dataset S1). After trimming, over 191 million good quality reads remained, of which over 155 million reads were mapped to the zebrafish genome. In all libraries, Piwi-interacting RNA (piRNA) were the most abundant RNA fraction, constituting from 31% (24 wpf testis) to 79% (unfertilized eggs) of all annotated small RNAs (Supplementary Fig. S1). Ovary and testis at 24 wpf had a comparatively large proportion of transfer RNA (tRNA, 24% and 31%, respectively). Large intergenic non-coding RNAs (lincRNAs) composed from 9% (24 wpf ovary) to 29% (spermatozoa) of small RNA reads. The proportion of miRNA greatly varied between libraries, ranging from 2 to 21% in unfertilized eggs and 24 wpf ovary, respectively.

The majority of small RNA reads in each library was between 26 and 31 nucleotides (nt) long (Supplementary Fig. S1). Libraries from undifferentiated 3 wpf gonads and gametes had a relatively large proportion of reads smaller than 20 nt. Libraries from gonads at 6 and 9 wpf generally showed few reads outside the 26 to 31 nt range. In contrast, libraries from 12 and 24 wpf gonads had strong peaks at 22 and 34 nt.

### Low input RNA protocol

**Figure 2:**
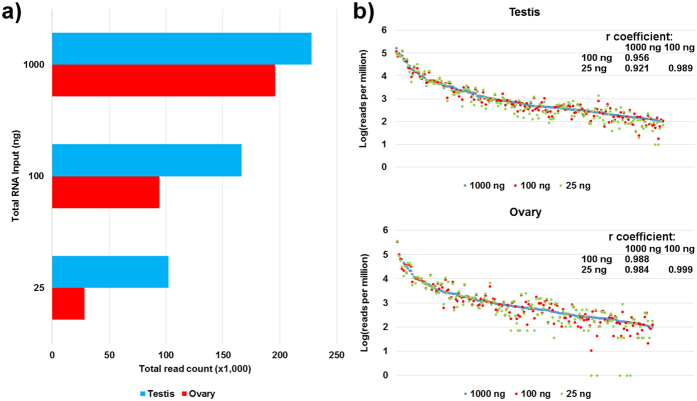
Efficiency of low-input RNA library preparation. (**a**) Total number of miRNA reads obtained from libraries prepared using different total RNA inputs. (**b**) Normalized miRNA reads from total RNA low-input libraries plotted against the data derived from the standard 1000 ng total RNA input library. Pearson’s product-moment correlation coefficient *r* is given for miRNAs with a threshold abundance >100 reads per million.

Total number of miRNA reads obtained from reduced RNA inputs were reduced to 73% and 45% in testis, and 48% and 14% in ovary, for libraries produced from 100 and 25 ng total RNA, respectively (Fig. 2a, Supplementary Dataset S2). Despite a reduction in the total number of miRNA reads, the frequency of mapped miRNAs in all RNA input levels was stable, as well as highly and positively correlated between input sizes (Fig. 2b). In 5 cases, individual miRNAs were not detected in the ovary library prepared from 25 ng total RNA input, while they were detected when using larger RNA inputs (Fig. 2b).

### Dynamics of miRNA abundance during gonadal development

**Figure 3:**
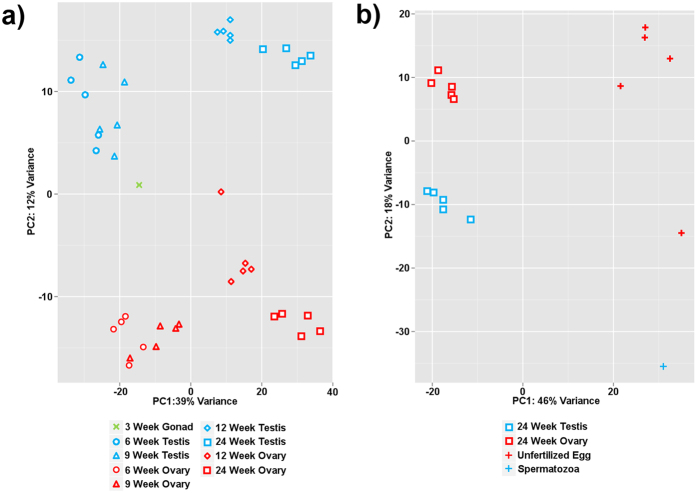
Principal component analysis of miRNAs from zebrafish gonads and gametes. (**a**) Clustering of miRNAs according to gonadal sex and developmental advancement. (**b**) Clustering of miRNAs in gametes versus respective gonad samples derived from sexually mature 24-week-old zebrafish. Blue and red colors denote male and female sex samples, respectively, while green color marks sexually undifferentiated 3-week-old juvenile gonad.

In total, 520 mature miRNAs were identified in zebrafish gonads (Supplementary Dataset S3). The samples were clustered according to sex (ovary versus testis) and developmental advancement (Fig. 3a). Samples from 6 and 9 wpf gonads were closely clustered within the respective sex, while 12 and 24 wpf gonad samples were clustered separately. The undifferentiated 3 wpf gonad sample was most similar to 6 and 9 wpf testis. A single ovary sample at 12 wpf was separated from the rest of the 12 wpf ovaries. When comparing gametes with 24 wpf gonads, the samples were tightly clustered as testis, ovary, unfertilized egg, and spermatozoa (Fig. 3b).

**Figure 4:**
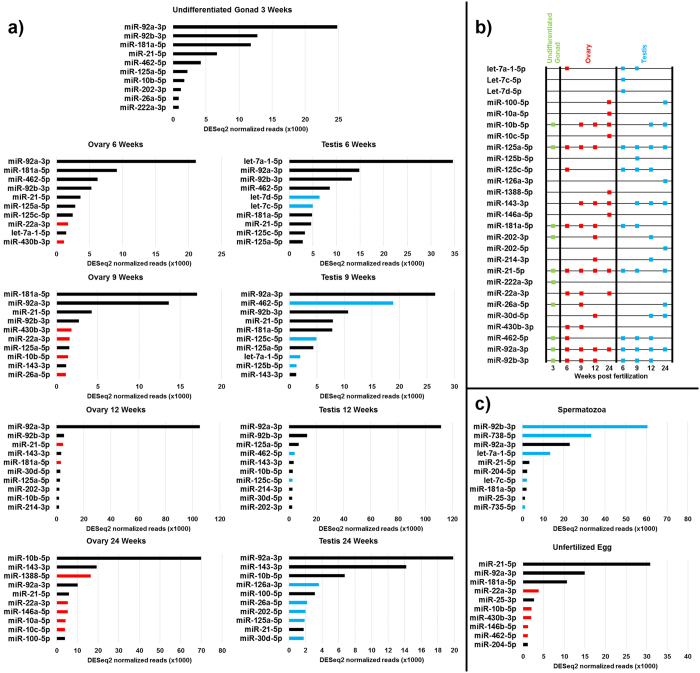
The most abundant miRNAs in zebrafish gonads and gametes. (**a**) The 10 most abundant miRNAs at each sampled stage of gonadal development in both testis and ovary. (**b**) The frequency of presence of a miRNA among the 10 most abundant miRNAs throughout the stages of gonadal development. (**c**) The 10 most abundant miRNAs in the spermatozoa and unfertilized eggs. Black bars represent miRNAs commonly abundant in both sexes at a given sampling time point, while red and blue bars mark miRNAs abundant in either ovary or testis samples, respectively.

Twenty-seven miRNAs contributed to the ten most abundant miRNAs in each gonadal sampling point (Fig. 4a,b). Five miRNAs were abundant only in purified spermatozoa or unfertilized eggs, but not in whole gonads (Fig. 4c). miR-92a-3p was the most frequently abundant miRNA, which was among the top five most abundant miRNAs in every gonadal stage as well as in gametes, while miR-92b-3p was highly abundant in gonads from 3 to 12 wpf, and in spermatozoa. In addition, miR-10b-5p, miR-125a-5p, miR-143–3p, miR-181a-5p, and miR-21–5p were among the most abundant miRNAs in at least six of nine gonadal stages (Fig. 4b). Several miRNAs, namely let-7a-1–5p, let-7c-5p, let-7d-5p, miR-181a-5p, miR-222a-3p, miR-430b-3p, and miR-462-5p were typically more abundant in earlier stages of gonadal development, whereas miR-100-5p, miR-10a-5p, miR-10b-5p, miR-10c-5p, miR-202-5p, and miR-30d-5p were abundant during later stages of development. miR-22a-3p and miR-430b-3p were only abundant in the ovary, whereas miR-125c-5p and miR-462-5p were more frequently abundant in the testis (6 to 12 wpf) compared to the ovary (only 6 wpf).

**Figure 5:**
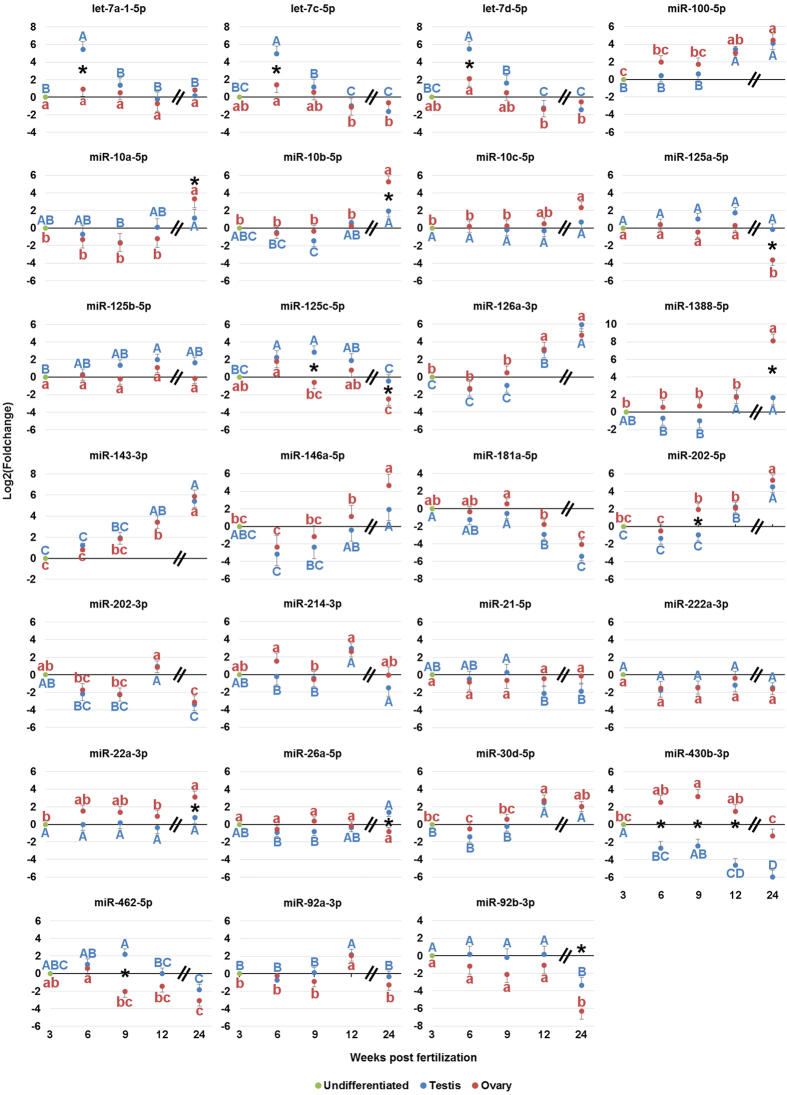
Dynamics of miRNA expression in zebrafish gonads over time. Each data point represents the average log-transformed fold change for the reads at a given time point (n = 5 for each testis and ovary sampling point) relative to undifferentiated gonads (pooled sample) at 3 weeks post fertilization. Within each sex, values marked with different letters (blue upper case for testis, red lower case for ovary) are significantly different from each other. Reads were normalized using DESeq2 and statistical significance was determined by an adjusted *p*-value ≤ 0.01 and a log2 fold change ≥ |2|. Significant differences between sexes at a given time point are marked with asterisks. The double break at X-axis highlights the discontinuous sampling interval between 12 and 24 weeks post fertilization. Vertical bars represent standard deviation.

Differential expression analysis of the dynamics of miRNA over the course of gonadal development revealed significant changes in miRNA abundance over time (Supplementary Dataset S4). Of the 520 mature miRNA identified in zebrafish gonads, 111 had at least one significant change in abundance over time. Abundance of 62 miRNAs in the testis and 65 miRNA in the ovary was significantly different from undifferentiated gonads in at least one sampling point. Additionally, 73 and 70 miRNAs had a significant change in abundance over time within the testis and ovary, respectively. The dynamics of expression of the 27 most abundant miRNAs (Fig. 4) had several distinct patterns (Fig. 5). miR-222a-3p was the only miRNA with no significant change in abundance over time, while miR-10a-5p, miR-10c-5p, miR-125a-5p, miR26a-5p, and miR-92b-3p showed no significant change in abundance from 3 to 12 wpf. Notably, all miRNAs from the miR-10 family showed a significant increase in abundance in ovaries at 24 wpf, while miRNAs from the let-7 family showed a significant increase at 6 wpf only. By comparison, miR-125c-5p, miR-430b-3p, and miR-462-5p all showed increase in abundance at 9 wpf. The abundance of several miRNAs, namely miR-100-5p, miR-126a-3p, miR-143-3p, and miR-202-5p was gradually increasing in time. In contrast, abundance of miR-181a-5p steadily decreased over time.

**Figure 6:**
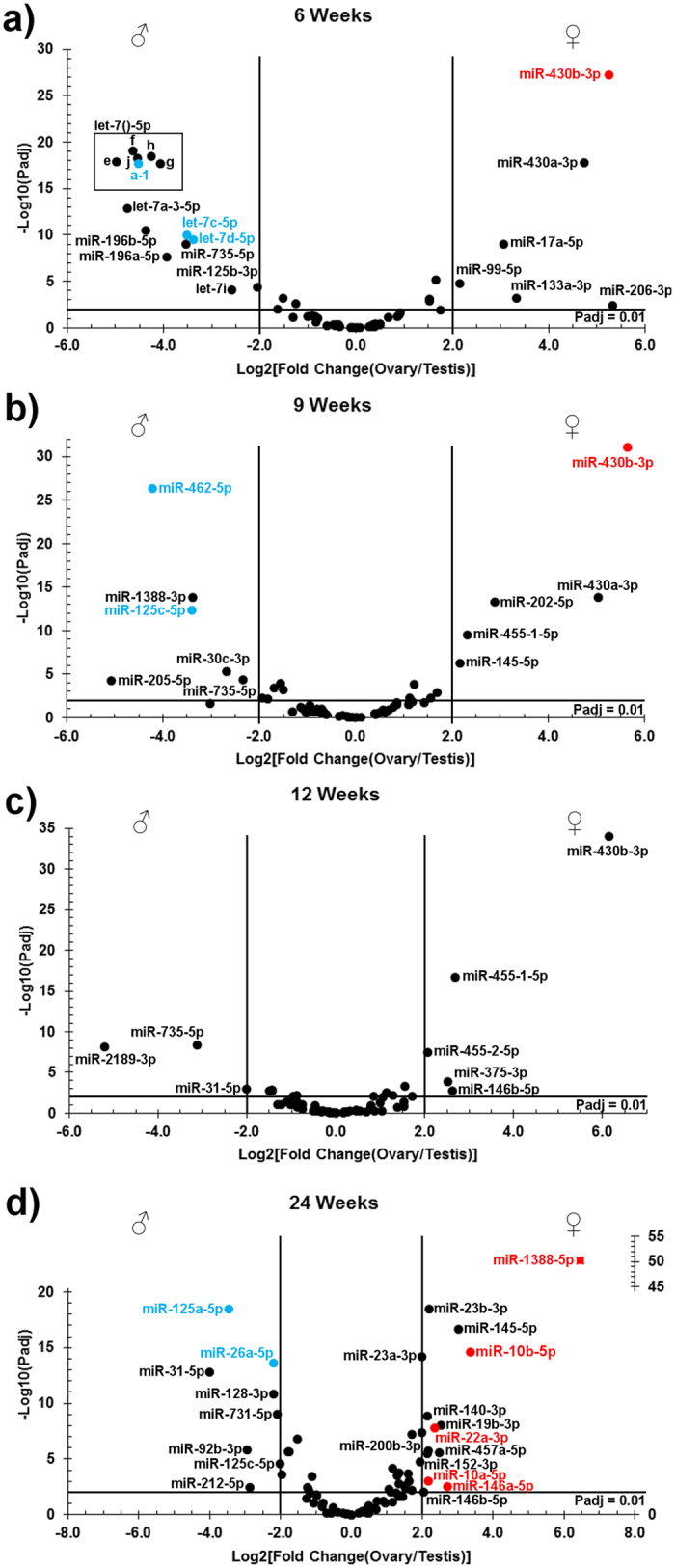
Differences in miRNA abundance between testes and ovaries. The plots show adjusted *p-*value (y-axis) against the log2-transformed fold change (ovary/testis) for miRNAs with a normalized read count ≥ 100 at 6 (panel a), 9 (**b**), 12 (**c**), and 24 weeks post fertilization (**d**). Named miRNAs appearing with a log2 fold change value ≥ |2| and an adjusted p-value ≤ 0.01 were considered as differentially expressed. Blue and red labels identify miRNA present among the 10 most abundant miRNA at the given time point for testis and ovary, respectively. The square data point in panel D (miR-1388-5p) is plotted against the secondary y-axis.

In total, 27 miRNAs were significantly more abundant in testis than in ovary in at least one developmental stage, whereas 23 miRNAs were more abundant in the ovary compared to the testis (Supplementary Dataset S5). Of the testis-enriched miRNAs, the let-7 family was well represented at 6 wpf (Fig. 6a), while the miR-125 family was abundant at 6, 9, and 24 wpf (Fig. 6a,b,d). miR-462-5p and miR-26a-5p were abundant at 9 and 24 wpf, respectively (Fig. 6b,d). In ovaries, miR-430b-3p was highly enriched compared to testes from 6 to 12 wpf (Fig. 6a,b,c). At 24 wpf, miR-10a-5p, miR-10b-5p, miR-146a-5p, miR-22a-3p, and miR-1388-5p were abundant and ovary-enriched (Fig. 6d).

### Comparison of miRNA abundance between gametes and sexually mature gonad

**Figure 7:**
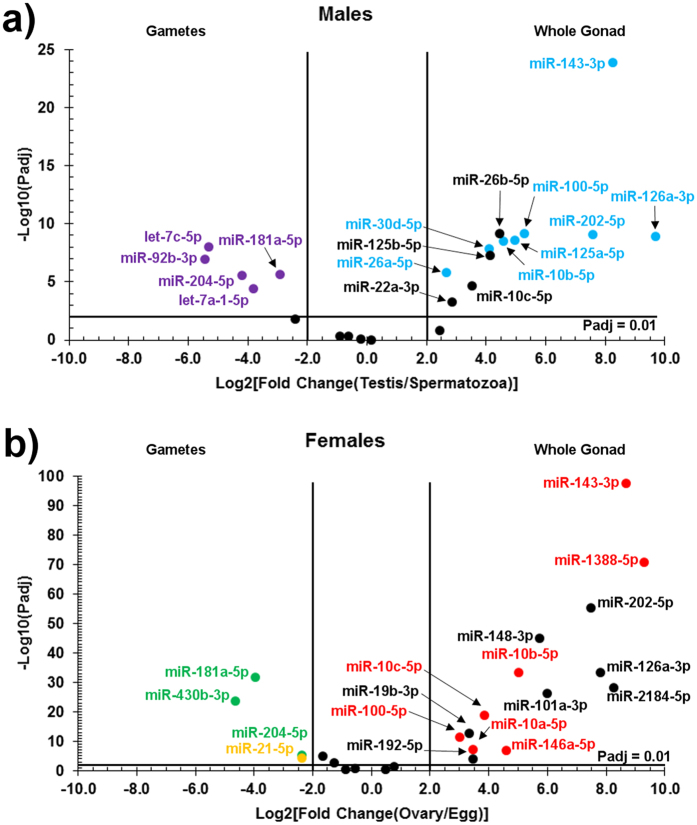
Differences in miRNA abundance between mature gonads and gametes. The adjusted *p*-value (y-axis) is plotted against the log- transformed fold change (gonad/gamete) for mature male (panel a) and female (**b**) zebrafish at 24 weeks post fertilization. Only miRNAs with a normalized read count ≥1000 are plotted. Named miRNAs appearing with a log2 fold change value ≥ |2| and an adjusted *p*-value ≤ 0.01 were considered differentially expressed. Colored labels identify miRNA among the 10 most abundant in their respective sample: blue for testis, red for ovary, purple for spermatozoa, green for unfertilized eggs. Yellow coloring identifies miRNA among the 10 most abundant in both ovaries and unfertilized eggs.

Comparing miRNA reads in gametes to the sexually mature gonads at 24 wpf, when both gametes and gonads were isolated, identified seven miRNAs with significantly increased abundance (Fig. 7, Supplementary Dataset S5). miR-181a-5p and miR-204-5p were significantly more abundant in both spermatozoa and oocytes. let-7a-1-5p, let-7c-5p and miR-92b-3p were more abundant in spermatozoa only, whereas miR-430b-3p and miR-21-5p were more abundant in oocytes only. miR-21-5p was the only miRNA which was among the most abundant miRNA in both unfertilized eggs and the ovary (Fig. 4a,c). By comparison, 20 miRNAs were significantly more abundant in gonads compared to gametes. The miR-10 family was well represented; miR-125a-5p and miR-125b-5p were more abundant in the testis than spermatozoa, miR-10a-5p was more abundant in the ovary than unfertilized eggs, while miR-10b-5p, miR-10c-5p and miR-100-5p were more abundant in both testes and ovaries compared to gametes.

### Presence of isomiRs during zebrafish gonadal development

**Figure 8:**
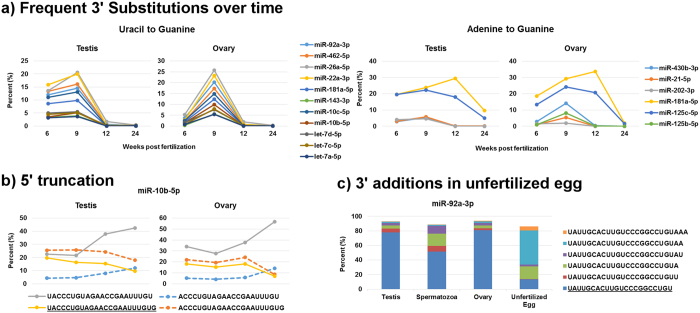
General features of isomiRs in zebrafish gonads. (**a**) Dynamics in the relative composition of frequently occurring nucleotide substitutions among the most abundantly expressed miRNAs in zebrafish gonads. (**b**) Representation of 5′ truncations observed in miR-10b-5p. Dotted lines represent isomiRs with 5′ truncation compared to the reference variant. (**c**) Abundant 3′ additions of templated adenine and untemplated uracil in miR-92a-3 in gametes versus mature whole gonads. The reference variants for miR-10b-5p (**b**) and miR-92a-3p (**c**) are underlined. Only isomiRs accounting for ≥5% of the total miRNA reads are shown.

Multiple variations in miRNA sequences (isomiRs) were observed throughout gonadal development (Supplementary Dataset S6). Of the 27 most abundant miRNAs from Fig. 4, all had at least one isomiR variant accounting for at least 5% of the miRNA reads in at least one developmental stage. Nucleotide additions were the most frequent variant, present in 23 out of the 27 miRNA examined (Supplementary Dataset S6). Of these, 13 miRNA had variants only containing templated additions, 4 miRNAs had only untemplated variants, and 6 miRNAs had both templated and untemplated variants. Nucleotide substitutions at 3′ end were observed in 18 of 27 miRNA (Supplementary Dataset S6). The most frequent substitutions were either uracil or adenine to guanine, found in 11 and 6 miRNAs, respectively (Fig. 8a). The U-to-G substitutions were frequently detected at 6 wpf in both testis and ovary, as well as 9 wpf in ovary, but not in 12 and 24 wpf gonads. The A-to-G substitutions were most frequent at 9 and 12 wpf. Modifications at 5′ end were detected in 8 miRNAs; miR-100-5p, miR-10a-5p, miR-10b-5p, miR-10c-5p, and miR-126a-5p were truncated, while miR-202-5p, miR-202-3p, and miR-214-3p had templated additions (Supplementary Dataset S6). In the case of miR-10b-5p, one of the 5′ truncated variants accounted for up to 26% of all reads, and was the dominant variant in the testis at 6 and 9 wpf (Fig. 8b). miR-202-3p had 10 variants with at least 5% representation, and the 5′ templated addition variants accounted for the majority of the reads in all developmental stages (Supplementary Dataset S6). In gametes, miR-92a-3p had several variants with 3′ templated adenine and untemplated uracil additions (Fig. 8c). In unfertilized eggs, isomiRs with an additional 1 to 3 adenine residues accounted for a combined 69% of all reads, compared to 7% in 24 wpf ovaries.

### GO term enrichment analysis

Gene ontogeny (GO) term enrichment analysis of the 27 most abundant gonadal miRNAs identified 16 significantly enriched biological process GO terms (Supplementary Dataset S7). The most enriched GO term was segment polarity determination (GO:0007367), which contained target mRNA for the miR-10 and miR-125 families, as well as miR-181a-5p miR-21-5p, miR-222a-3p, and miR-430b-3p. The second most enriched GO term was negative regulation of substrate adhesion-dependent cell spreading (GO:1900025), and contained target mRNA for miR-181a-3p, miR-202-5p, miR-25-3p, miR-30d-5p, miR-430b-3p, miR-92a-3p, and miR-92b-3p.

## Discussion

A comparison of the zebrafish miRNA repertoire in sexually mature gonads with sequencing results in olive flounder (*Paralichthys olivaceus*)^33^, marine medaka (*Oryzias melastigma*)^34^, Nile tilapia^26^, rainbow trout^22^, and another zebrafish study^27^, identified several miRNAs with varying levels of gonadal conservation, while highlighting the dynamic nature of miRNA expression (Supplementary Fig. S2). Among the most frequently abundant miRNAs between species were let-7a-5p, miR-143-3p, miR-181a-5p, miR-202-5p, and miR-21-5p (Supplementary Fig. S2B). The most frequently abundant was miR-143-3p, which was among the 10 most abundant miRNAs in both testis and ovary in all five species examined, followed by miR-21-3p, which was abundant in all except Nile tilapia. Relative abundance of let-7a-5p, miR-181a-5p, and miR-202-5p varied between different species as well as the two zebrafish studies. We detected high abundance of let-7a-5p in testis at 6 wpf (Fig. 4), but not in sexually mature gonads, in contrast to the other four fish species, and the other zebrafish study^27^. miR-202-5p had high abundance in rainbow trout, marine medaka, and the other zebrafish study, but we detected comparatively lower abundance (Fig. 4), similar to Nile tilapia. miR-181a-5p was highly abundant in Nile tilapia and olive flounder, as well as 12 wpf zebrafish ovaries (Fig. 4), but not highly abundant at 24 wpf ovary or in the related zebrafish study.

We report relatively high precision in small RNA sequencing between biological replicates, allowing us to detect significant differences in abundance between the gonadal stages sampled (Fig. 5, Supplementary Dataset S4). However, we also observed changes in the miRNA repertoire between 12- and 24-week-old gonads, both of which were sexually mature, but differed in gonadal morphology (Fig. 1). These findings suggest small RNA sequencing at a single developmental point may be inadequate to reliably determine the miRNA transcriptome of a gonad.

Since zebrafish germline *dicer* and *ago2* mutants are fertile^17^,^18^, and germline miRNA activity in mice is suppressed during oocyte maturation^15^, there are questions over the essentiality of miRNAs in gonadal development. By comparison, piRNA and Piwi proteins are essential to zebrafish gonadal development^19^,^20^. Zebrafish *ziwi* mutants have normal gonadal development at 2 wpf, before suffering severe germ cell depletion by 3 wpf as a result of apoptosis^20^. In *zili* mutants, a nonsense mutation resulted in complete germline ablation after 7 wpf, while a missense mutant resulted in germ cell development but caused defects during meiosis I in oocytes and ended in sterility^19^. Between 2 and 3 wpf, wildtype zebrafish germ cells are proliferating and entering the early stages of meiosis prior to sex determination^29^, while the number of germ cells produced influences the eventual sex of the animal^35^,^36^. In our study, we observed that the miRNAs abundant in 3 wpf gonads generally maintained steady levels of expression throughout the gonadal development, and had putative functions in repressing stem cell differentiation, notably: miR-10b-5p, miR-222a-3p, and miR-26a-3p (Figs 4 and 5). miR-10b-5p belongs to the evolutionarily conserved miR-10/100 family, which are associated with suppressing *homeobox (hox*) genes^37^; *hox* genes are highly conserved transcription factors which guide stem cell differentiation^38^. In mice, miR-10b-5p was enriched in germ cells, and suggested to function in repressing germ cell differentiation^39^. In chicken (*Gallus gallus*), miR-222-3p is germline enriched and represses *de novo methyltransferase (DNMT3B*)^40^. In the olive flounder, miR-26a-5p targets *empty spiracles homeobox 2 (emx2*)^41^; in mice, *Emx2* is required to trigger germ cell differentiation^42^. These findings suggest there may be a relationship between miRNAs functioning to repress early gonadal cell differentiation, while piRNAs are required for germ cell differentiation, an initiation of meiosis, and to prevent germ cell apoptosis.

The role of gonadal miRNAs may also be more essential to gonadal somatic cell development rather than to the germline. In this study, while miRNAs made up over 20 percent of all small RNA reads in gonads at 24 wpf, they made up less than 3 percent of small RNA reads in isolated gametes (Supplementary Fig. S1). In addition, we found far more miRNAs with significantly higher abundance in whole 24 wpf gonads compared to isolated gametes (Fig. 7). While germline *dicer* and *ago2* mutations effectively prevented mature miRNAs transcription within the zebrafish germline^17^,^18^, they would not have prevented miRNA expression in gonadal somatic cells. In mice, Dicer loss in Sertoli cells led to infertility^13^, with evidence suggesting the altered proteome was a result of miRNA depletion^43^. While it is possible that miRNAs are more abundant within the germline in earlier stages of gametogenesis than in mature gametes, the differences we observed were striking and should be investigated further. *In situ* hybridization experiments identifying spatial expression patterns of miRNAs would assist in determining whether they have primary functions in the germline, gonadal somatic cells, or both.

In contrast to the miRNAs abundant at 3 wpf, many of the miRNAs abundant later in gonadal development had putative functions in promoting cell differentiation. We found significantly increased abundance of let-7 miRNAs in testis at 6 wpf (Fig. 5). At this developmental stage, the gonad is already differentiated (Fig. 1) and typically consists of spermatogonia and spermatocytes with some spermatids^29^. The let-7 miRNA family is highly conserved and typically associated with promoting progenitor cell differentiation and proliferation, often through interactions with *lin41/trim71* and *lin28*^44^. Lin28 interacts with pre-let-7 to prevent its maturation, which results in cells maintaining an undifferentiated state. Trim71 promotes Lin28 repression of let-7 and antagonizes Argonaute 2 (Ago2), resulting in protection of let-7-targeted mRNA from repression^44^,^45^,^46^. miRNAs from the let-7 family target *trim71*, meaning increased abundance of let-7 causes a positive feedback loop of let-7 maturation and function, thereby encouraging progenitor cell differentiation^44^,^45^,^46^. In mouse, Trim71 is present within the male germline, and let-7a promotes spermatogonia differentiation^47^. However, in fruit fly (*Drosophila melanogaster*), let-7 promotes germline differentiation through regulating gonadal-somatic cell behaviour^48^. The specific timing of let-7 abundance observed in zebrafish testis suggests a conserved function in promoting testis differentiation, although future studies will be needed to determine whether teleost let-7 functions primarily through the germline or gonadal somatic cells.

Notable miRNAs exhibiting a pattern of increasing abundance throughout the gonadal development included miR-100-5p, miR-126a-3p, and miR-143-3p (Fig. 5), all of which also have putative functions in promoting cell differentiation. In human, miR-100-5p promotes stem cell differentiation and impairs the self-renewing ability of breast cancer stem cells^49^, while miR-126-3p was suggested to function in the differentiation and morphology of PGCs^39^,^50^. Several studies have shown mammalian miR-143-3p to function in suppressing cell proliferation^51^,^52^, regulating cell apoptosis^53^, and regulating differentiation of cultured adipocyte cells^54^.

miR-92a-3p was the most dominant gonadal miRNA in our study (Fig. 4). While miR-92a-3p abundance at 12 wpf was overwhelming, our observations were similar to studies in the mature gonads of zebrafish and yellow catfish (*Pelteobagrus fulvidraco*)^24^,^27^. The miR-17/92 cluster is well conserved and has been linked to regulating the cell cycle, proliferation, and apoptosis^55^. In a previous study, we demonstrated that the knockdown of miR-92a-3p in zebrafish gonads resulted in significantly reduced miR-92a-3p expression in 1-cell stage embryos, which also had a significantly higher rate of embryonic developmental arrest^29^. This led us to hypothesize miR-92a-3p had a function as a maternal miRNA in zebrafish embryogenesis. Here, we found that an exceptionally large proportion of miR-92a-3p transcripts in unfertilized egg, and to a lesser extent spermatozoa, had templated 3′ nucleotide additions (Fig. 8c). Maternal miRNAs are highly adenylated in oocytes and early embryos of fruit fly, sea urchin (*Strongylocentrotus purpuratus*), and mouse^56^, with the adenylation functioning to regulate miRNA stability^57^,^58^,^59^. Our findings further suggest miR-92a-3p is stored as a maternal transcript in unfertilized eggs.

At present, relatively little is known about how maternal miRNAs function in zebrafish embryos. The most studied embryonic miRNA in zebrafish is miR-430-3p, which is transcribed before the maternal to zygotic transition and functions as part of the zygotic decay pathway to destabilize maternal mRNAs^60^. However, zebrafish miR-430-3p only targets a relatively small number (~10%) of maternal mRNAs^60^, and many maternal mRNAs are instead regulated through a maternal decay pathway^61^,^62^. Recently, studies of the maternal decay pathway in zebrafish identified the presence of uncommon codons, such a leucine, and short 3′UTRs as important determinants in maternal mRNA stability^63^. In the future, it would be interesting to examine if there is a correlation between predicted targets of maternal miRNAs and these attributes.

Several miRNAs were consistently more abundant in testis than ovary, including miR-125a-5p, miR-125b-5p, miR-125c-5p, and miR-462-5p, whereas miR-22a-3p and miR-430b-3p were more abundant in the ovary (Fig. 6). Our findings were similar to deep sequencing results in mature zebrafish gonads, which also reported higher abundance of 125a-5p, miR-125b-5p, miR-125c-5p, and miR-462-5p in the testis, although that study did not find higher abundance of miR-22a-3p in the ovaries^27^. In mammals, miR-22 was shown to regulate estrogen receptor α^64^, which acts as a transcription factor to regulate reproductive development^65^. Neither our study, nor the study by Vaz *et al*.^27^ found miR-430b-3p to be abundant in mature ovaries. However, we did find miR-430b-3p to be among the most abundant miRNA in ovulated oocytes (Fig. 4c). A previous study on zebrafish ovarian development found miR-430b-3p to be differentially expressed in oocyte and follicular cells^66^. In follicular cells, miR-430b-3p expression was highest in early vitellogenic stages before being significantly reduced during late vitellogenesis and maturation, whereas its expression in oocytes was highest during late vitellogenesis and was still strongly expressed in mature oocytes. Among the predicted targets of miR-430b-3p are *inhibin beta Aa (inhbaa*), a subunit of Activin, *activin-A receptor type II-like (acvrl1*), as well as *smad3a* and *smad3b*^67^. Activin, which is essential to zebrafish follicle development and ovary maturation^68^,^69^, is an extracellular signaling ligand which activates the intracellular Smad signaling pathway upon binding with its type-II receptor^70^. Luteinizing hormone, which up-regulates activin A production in ovarian follicles to induce maturation^71^, also inhibits miR-430b-3p expression within the follicles^66^, suggesting miR-430b-3p may act within follicle cells to suppress ovary maturation.

The testis-enriched miRNAs, found in our study, share similar putative functions as regulators of cell proliferation and apoptosis, including miR-125a-5p and miR-125b-5p^72^,^73^,^74^. Additionally, in mouse it was shown that Dicer, which is required for miRNA biogenesis, is essential for Sertoli cell survival and subsequent germ cell development^13^. Sertoli cells with a Dicer-knockout mutation lacked several miRNAs, including miR-125a-3p, while having an overabundance of SOD-1, a protein linked to apoptotic cell death^43^. In zebrafish embryos, miR-462–5p was also shown to regulate cell proliferation and apoptosis^75^.

In the present study, we observed a shift in the dominant miR-202 arm from 3p to 5p between 12 and 24 wpf (Figs 3 and 4). Our findings are in agreement with other studies on teleost, which found miR-202-5p to be the dominant arm in the mature gonads of zebrafish^27^ and tilapia^26^, while miR-202-3p was the dominant arm in the immature gonads of Atlantic halibut^76^. Arm switching in miRNA has been documented in mammals, where the dominant arm of several miRNA varied between tissues and greatly increases the number of regulatory targets of a pre-miRNA^77^. For miR-202, the observed arm-switch may be related to different arm preference between gonadal-somatic and germline cells. In mouse, both 5p and 3p arms were expressed in embryonic gonadal-somatic cells but not cells of the germline^78^. However, in frog (*Xenopus tropicalis*), miR-202-5p was detected in oocytes during oogenesis^79^.

Currently, the function of miR-202 in teleosts is unknown, and as such, it is difficult to explain the shift in arm preference between developmental stages. In chicken and mouse, miR-202 is enriched in testis and pri-miR-202 was suggested to be a direct transcriptional target of SOX9/SDF1 and involved in promoting testis differentiation^78^,^80^,^81^. However, in zebrafish, neither miR-202-5p nor miR-202-3p were found to be enriched in testis compared to ovary, a finding which is supported by another recent zebrafish study^27^. Similarly, in rainbow trout, both 5p and 3p arms were present in ovaries during late vitellogenesis^23^, where target prediction suggested miR-202-5p targets several miRNAs essential to oogenesis, such as *transforming growth factor β receptor II (tgfbr2*)^23^. In mouse, *Tgfbr2* is required to maintain primordial follicle quiescence, and its suppression leads to the initiation of ovarian primordial follicle development^82^. In zebrafish ovaries, the follicle layer is formed by 9 wpf^83^, which coincides with the significant increase in miR-202-5p abundance observed in our study. miR-202-3p in human has been shown to function in suppressing cell proliferation^84^,^85^. In the present study, we found miR-202-3p to be among the most abundant gonadal miRNA at 3 and 12 wpf (Fig. 4). High abundance at 3 wpf may suggest miR-202-3p functions in maintaining the state of the undifferentiated gonad, while high abundance at 12 wpf, when zebrafish gonads are reaching sexual maturity, may be related to the meiotic arrest of gametes.

Among the isomiRs detected in zebrafish gonads, 3′ substitutions of either uracil or adenine to guanine were frequently found at 6 and 9 wpf (Fig. 8a). Adenine-to-inosine RNA editing has previously been documented as a widespread post-transcriptional modification of miRNA precursors^86^,^87^. As inosine resembles guanosine, it would appear in sequencing as an A to G substitution and explain our observations. To our knowledge, no mechanisms for uracil to guanine or inosine substitutions have been described and false positives in RNA editing are a known bias of RNA sequencing^88^. We attempted to validate isomiRs using Sanger sequencing but it was unsuccessful (data not presented), likely due to amplification of the dominant variant over lesser represented isomiRs. Regardless, the frequency of uracil-to-guanine substitutions was striking, particularly in ovary at 9 wpf, where in the case of miR-26a-5p it exceeded 25% of the miRNAs’ reads (Fig. 5). In mouse, isomiRs with guanine at the 3′ terminus preferentially localize to the nucleus of neurons^89^. The authors suggested the 3′ terminal guanine may have higher stability in the nucleus, or the 3′ guanine may mediate active transport to the nucleus through interaction with Argonaute proteins. It was also suggested that altering the sub-cellular localization of a miRNA could result in distinct functions at particular developmental stages.

Trimming variants at the 5′ and 3′ ends of miRNAs have been previously reported^90^,^91^. The 5′ end change can affect the seed sequence and alter the function of a miRNA^90^. In our study, we detected several miRNAs with either templated additions or truncations at the 5′ end, notably in the miR-10 family and in both miR-202-5p and 3p arms (Fig. 8b, Supplementary Dataset S6). While we were unable to validate these isomiRs, a 5′ trimming variant of miR-202-5p with a similar relative proportion of reads was also detected in the testis and ovary of marine medaka^34^.

The zebrafish used in the experiment are the hybrid *See-Thru-Gonad* line^29^. To date, there have been no studies comparing the miRNA repertoire between zebrafish lines, and we therefore cannot rule out mutations or the eGFP transgene affecting gonadal miRNA expression. However, a previous study which used the *nacre transparent* (−/−), zf45Tg, and TAB reference line found no morphological differences in gonadal development between them^92^.

In summary, this study reports the miRNA repertoire in zebrafish gonads throughout the development from undifferentiated gonads to sexual maturation. We have demonstrated the dynamic changes in miRNA abundance at key stages of gonadal development. Our data show various trends in miRNA abundance, with several miRNAs consistently among the most abundant miRNAs in all gonadal stages, whereas others show specific time or sex dependent expression. The reported miRNA abundance patterns also often correlated to the putative miRNA functions. This is the first report on the dynamics of small RNA transcriptome in both testis and ovary in a teleost, and therefore provides the most complete overview of changes in miRNA abundance in gonads over time. The results from this study will serve as an important platform from which future studies can begin to validate the functional roles of gonadal miRNAs.

## Methods

### Fish

All experiments involving zebrafish were performed according to the Norwegian Regulation on Animal Experimentation (The Norwegian Animal Protection Act, No. 73 of 20 December 1974). All methods were approved by the National Animal Research Authority (Utvalg for forsøk med dyr, forsøksdyrutvalget, Norway) General License for Fish Maintenance and Breeding (Godkjenning av avdeling for forsøksdyr) no. 17.

The zebrafish used in the experiment were the *See-Thru-Gonad* line, previously established by crossing the *nacre transparent* (−/−)^31^,^32^ and zf45Tg lines^30^. The resulting hybrid line is transparent with a fluorescently labeled germline throughout development^29^.

Zebrafish were housed in a recirculating system (Pentair, Apopka FL, USA) and maintained according to standard procedures^93^. The system temperature was maintained at 28.5 ± 1.0 ˚C with a 12 h light and 12 h dark photoperiod. Adult zebrafish were housed in 10 L tanks at a density of 40 fish/tank with an approximate 1:1 sex ratio. When spawning, the fish were given a daily diet of newly hatched *Artemia* nauplii (Pentair) and SDS 400 zebrafish specific diet (Special Diet Services, Essex, United Kingdom). Juvenile zebrafish were produced using standard breeding techniques^93^. Post hatch, zebrafish larvae were kept in 3 L tanks with fine mesh baffles and restricted water flow. Feeding began at 2 days post hatch using SDS 100 zebrafish specific diet. From 2 to 4 weeks post fertilization (wpf) the juvenile fish were fed newly hatched *Artemia* nauplii and SDS 200 diet. After 4 wpf, the juveniles were fed SDS 300 diet alongside *Artemia* nauplii until the end of the experiment.

### Sampling

Gonads from zebrafish were sampled at 5 time points: 3, 6, 9, 12, and 24 weeks post fertilization (Fig. 1). For each time point, five individuals of each sex were euthanized using 200 mg/L tricaine methanesulphonate (MS-222; Sigma Aldrich, Oslo, Norway) buffered with equal parts sodium bicarbonate, followed by partial decapitation once opercular movement had ceased. Zebrafish were submerged in a Petri dish of ice-cold nuclease-free phosphate-buffered saline (PBS; Sigma Aldrich) where they were measured for total length using an AxioZoom V.16 microscope (Carl Zeiss Microscopy GmbH, Göttingen, Germany) equipped with an AxioCam MRm monochrome camera (Zeiss) and Zen Pro imaging software. Images were taken using either bright field or epifluorescent light with an enhanced green fluorescent protein filter (excitation: 488 nm, emission: 509 nm). Epifluorescent light was used to identify the gonad, which was subsequently removed and examined to determine the sex. The gonads were transferred to RNA Later (Sigma Aldrich) and kept on ice until RNA extraction.

In addition to the whole gonads, sampling of spermatozoa and unfertilized oocytes was performed. For spermatozoa, 24 wpf adult male zebrafish were immobilized by immersion in chilled Hanks’ balanced salt solution (Sigma Aldrich) following standard protocol for zebrafish cold-water immobilization^94^. The immobilized fish were patted dry with tissue paper before gently stroking the side of the fish. Sperm was collected from the genital pore using a 10 μL pipette with gentle suction. Spermatozoa from 15 fish was pooled in 200 μL ice-cold Hanks’ balanced salt solution before proceeding. After pooling, the suspension was centrifuged at 2500 g for 10 min, resulting in a spermatozoa pellet. The supernatant was discarded and the pellet was re-suspended in 200 μL of chilled Hanks solution. A 10 μL sub-sample was checked for spermatozoa mobility and presence of non-sperm cell contaminants using the AxioZoom V.16 microscope. The remaining spermatozoa were pelleted a second time as previously described and the supernatant was discarded. The pellet was immediately taken for RNA extraction. Unfertilized eggs were collected from five females using a similar method, except the fish were first immobilized in chilled PBS. After the fish were patted dry, unfertilized eggs were collected into a plastic spoon and maintained as individual batches for each female separately. After collection, the unfertilized eggs were transferred to a Petri dish and washed three times in ice-cold PBS before being taken immediately for RNA extraction.

### RNA extraction

Total RNA was extracted using QIAzol Lysis Reagent (Qiagen, Hilden, Germany) following manufacturer’s instructions. Following ethanol precipitation, the extracted RNA was purified using the RNeasy MinElute Cleanup Kit (Qiagen). During column purification, genomic DNA was removed using a DNase I treatment (Qiagen). RNA integrity was assessed and quantified using the Agilent 2200 Tapestation instrument (Agilent, Santa Clara, USA) using High Sensitivity RNA screen tape (Agilent).

### Small RNA library preparation, low-input RNA protocol optimization, and sequencing

Small RNA libraries were produced using TruSeq Small RNA Library Preparation kit (Illumina, San Diego, USA) following the manufacturer’s protocol with modifications to down-scale input RNA. All whole gonad and unfertilized egg libraries were produced from individual fish, whereas the spermatozoa library input was pooled from 15 individuals. Because total RNA yields from immature zebrafish gonads are not sufficient for the recommended 1000 ng input, the following modifications were made: when using 100 or 25 ng total RNA inputs, both the 5′ and 3′ ligation adapters were diluted 2x before use, and the number of amplification cycles used was 15, compared to 13 when using 1000 ng total RNA input. Adapter-ligated constructs were selected with a size of 145 to 170 nt. To test the efficacy and reliability of low-input RNA library preparation, RNA from a 24 wpf ovary and testis were extracted and libraries were prepared using inputs of 1000 ng, 100 ng, and 25 ng.

Adult 24 wpf gonads were sequenced using the recommended minimum input of 1000 ng total RNA. Ovary libraries from 6 to 12 wpf were prepared using 100 ng of total RNA. For testis libraries, 9 and 12 wpf libraries were prepared using inputs of 100 ng total RNA. Inputs of 25 ng total RNA were used for the 3 wpf undifferentiated gonads and 6 wpf testis samples. For unfertilized eggs and spermatozoa libraries, inputs of 1000 and 100 ng total RNA were used, respectively. Sequencing was performed using the MiSeq Reagent Kit v3 (150 cycles with single reads; Illumina) on a MiSeq.

### Data analysis

Adapter sequences and low quality (Phred quality score < 20) sequences were removed using cutadapt^95^. Trimmed good quality sequences were mapped to known zebrafish non-coding RNAs from Ensembl, GRCz10 (http://www.ensembl.org). miRDeep2^96^ was used to identify mature zebrafish miRNAs with single mismatch using miRBase version 21 (http://www.mirbase.org). The expression levels of mature miRNAs and differential expression were assessed using DESeq2 algorithm^97^. To be considered differentially expressed, miRNAs from comparable developmental stages required a minimum of 100 DESeq2 normalized reads in one stage, a Log2 (Fold change) value ≥ |2|, as well as an adjusted p-value ≤ 0.01. The normalization factor was estimated by taking the median of the ratio of a sample read count divided by the geometric mean across all samples^97^.

Zebrafish 3′UTRs and miRNAs were downloaded from ensembl 87^98^ and miRBase 21^99^ respectively. miRNA targets were predicted using miRanda^100^ with a minimum total alignment score of 155 and maximum free energy −20 kcal/mol. GO term enrichment analysis was performed for the 27 most abundant miRNAs (Fig. 4) using the empirical sampling approach^101^.

## Additional Information

**How to cite this article**: Presslauer, C. *et al*. Dynamics of miRNA transcriptome during gonadal development of zebrafish. *Sci. Rep.* **7**, 43850; doi: 10.1038/srep43850 (2017).

**Publisher's note:** Springer Nature remains neutral with regard to jurisdictional claims in published maps and institutional affiliations.

## Supplementary Material

Supplementary Figures

Supplementary Dataset 1

Supplementary Dataset 2

Supplementary Dataset 3

Supplementary Dataset 4

Supplementary Dataset 5

Supplementary Dataset 6

Supplementary Dataset 7
