# Supplementary material for: Dynamics of miRNA transcriptome during gonadal development of zebrafish
Source: Sci Rep. 2017 Mar 6;7:43850. doi: 10.1038/srep43850 (PMC5338332; doi:10.1038/srep43850)
Supplement: Supplementary Figures [file srep43850-s1.pdf]

Supplementary Files

Dynamics of miRNA transcriptome during gonadal development of zebrafish

Christopher Presslauer, Teshome Tilahun Bizuayehu, Martina Kopp, Jorge M.O. Fernandes, Igor Babiak

Supplementary Figure S1. Read numbers and distribution

(A) The total number of mapped small RNA reads and their class distribution. (B) Length distribution.

Supplementary Figure S2. Comparison of gonadal miRNA datasets between teleost species

(A) The number of miRNA common between mature gonadal miRNA datasets for olive flounder [1], marine medaka [2], Nile tilapia [3], and rainbow trout [4], as well as zebrafish from the present study at both 12 and 24 weeks post fertilization (wpf), and an additional study in zebrafish [5]. The graph was produced using UpSet [6]. (B) A comparison of commonly abundant gonadal miRNAs between teleost species and their frequency of presence in each dataset.

Supplementary Dataset S1. Sequence statistics

(Tab 1) The number of reads produced before and after quality trimming in each small RNA library. (Tab 2) The total number of reads mapped to each class of RNA. (Tab 3) The distribution of reads for each library by nucleotide length.

Supplementary Dataset S2. Low input RNA test

The absolute number of reads produced for individual miRNAs between different total RNA inputs used during small RNA library preparation.

Supplementary Dataset S3. miRNA reads for mapped miRNAs in each small RNA library
The absolute (Tab 1) and DESeq2 normalized (Tab 2) number of reads for all miRNAs in each library.

Supplementary Dataset S4. Differentially expressed miRNAs over time
Each tab displays differential expression analysis of DESeq2 normalized reads for different time comparisons. All testis stages are compared to each other as well as 3 week undifferentiated gonads. All ovary stages are compared to each other as well as 3 weeks undifferentiated gonads.

Supplementary Dataset S5. Differentially expressed miRNAs between sexes
Each tab displays differential expression analysis of DESeq2 normalized reads at each developmental stage between testis and ovary, as well as between gametes and sexually mature gonads at 24 weeks post fertilization.

Supplementary Dataset S6. IsomiRs in zebrafish gonads
List of the relative percentage for each isomiR within the 27 most abundant zebrafish gonadal miRNAs (Tab 1) and all sequenced reads for each isomiR detected (Tab 2).

Supplementary Dataset S7. miRNA target prediction and GO analysis
(Tab 1) Target prediction using miRanda v. 21 for the 27 abundant miRNAs in zebrafish gonads. (Tab 2) GO enrichment analysis for the 27 abundant miRNAs.

Supplementary References

1. Gu, Y., Zhang, L. & Chen, X. Differential expression analysis of *Paralichthys olivaceus* microRNAs in adult ovary and testis by deep sequencing. *Gen. Comp. Endocrinol.* **204**, 181-184, doi:10.1016/j.ygcen.2014.05.019 (2014).
2. Lau, K. *et al.* Identification and expression profiling of microRNAs in the brain, liver and gonads of marine medaka (*Oryzias melastigma*) and in response to hypoxia. *PLoS ONE* **9**, e110698, doi:10.1371/journal.pone.0110698 (2014).
3. Xiao, J. *et al.* Identification and characterization of microRNAs in ovary and testis of Nile tilapia (*Oreochromis niloticus*) by using solexa sequencing technology. *PLoS ONE* **9**, e86821, doi:10.1371/journal.pone.0086821 (2014).
4. Juanchich, A. *et al.* Characterization of an extensive rainbow trout miRNA transcriptome by next generation sequencing. *BMC Genomics* **17**, 164, doi:10.1186/s12864-016-2505-9 (2016).
5. Vaz, C. *et al.* Deep sequencing of small RNA facilitates tissue and sex associated microRNA discovery in zebrafish. *BMC Genom.* **16**, 1-16, doi:10.1186/s12864-015-2135-7 (2015).
6. Lex, A., Gehlenbord, N., Strobelt, H., Vuillemot, R. & Pfister, H. UpSet: visualization of intersecting sets. *IEEE Trans. Vis. Comput. Graph.* **20**, 1983-1992, doi:10.1109/tvcg.2014.2346248 (2014).

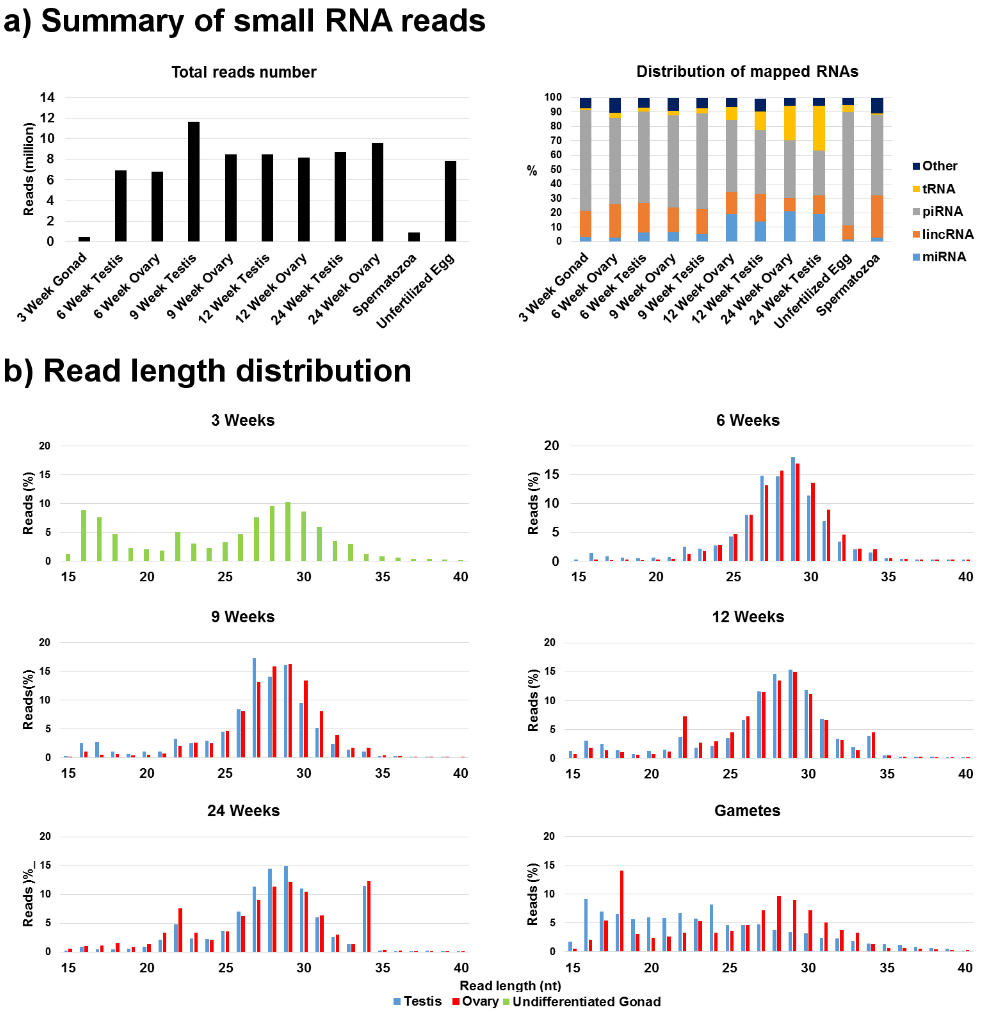
a) Summary of small RNA reads
Total reads number
Reads (million)
0
2
4
6
8
10
12
14
3 Week Gonad
6 Week Testis
6 Week Ovary
9 Week Testis
9 Week Ovary
12 Week Testis
12 Week Ovary
24 Week Testis
24 Week Ovary
Spermatozoa
Unfertilized Egg
Distribution of mapped RNAs
%
0
10
20
30
40
50
60
70
80
90
100
3 Week Gonad
6 Week Ovary
6 Week Testis
9 Week Ovary
9 Week Testis
12 Week Ovary
12 Week Testis
24 Week Ovary
24 Week Testis
Unfertilized Egg
Spermatozoa
Other
tRNA
piRNA
lincRNA
miRNA
b) Read length distribution
3 Weeks
6 Weeks
9 Weeks
12 Weeks
24 Weeks
Gametes
Reads (%)
Reads(%)
Reads)%_
0
5
10
15
20
15
20
25
30
35
40
Read length (nt)
Testis
Ovary
Undifferentiated Gonad

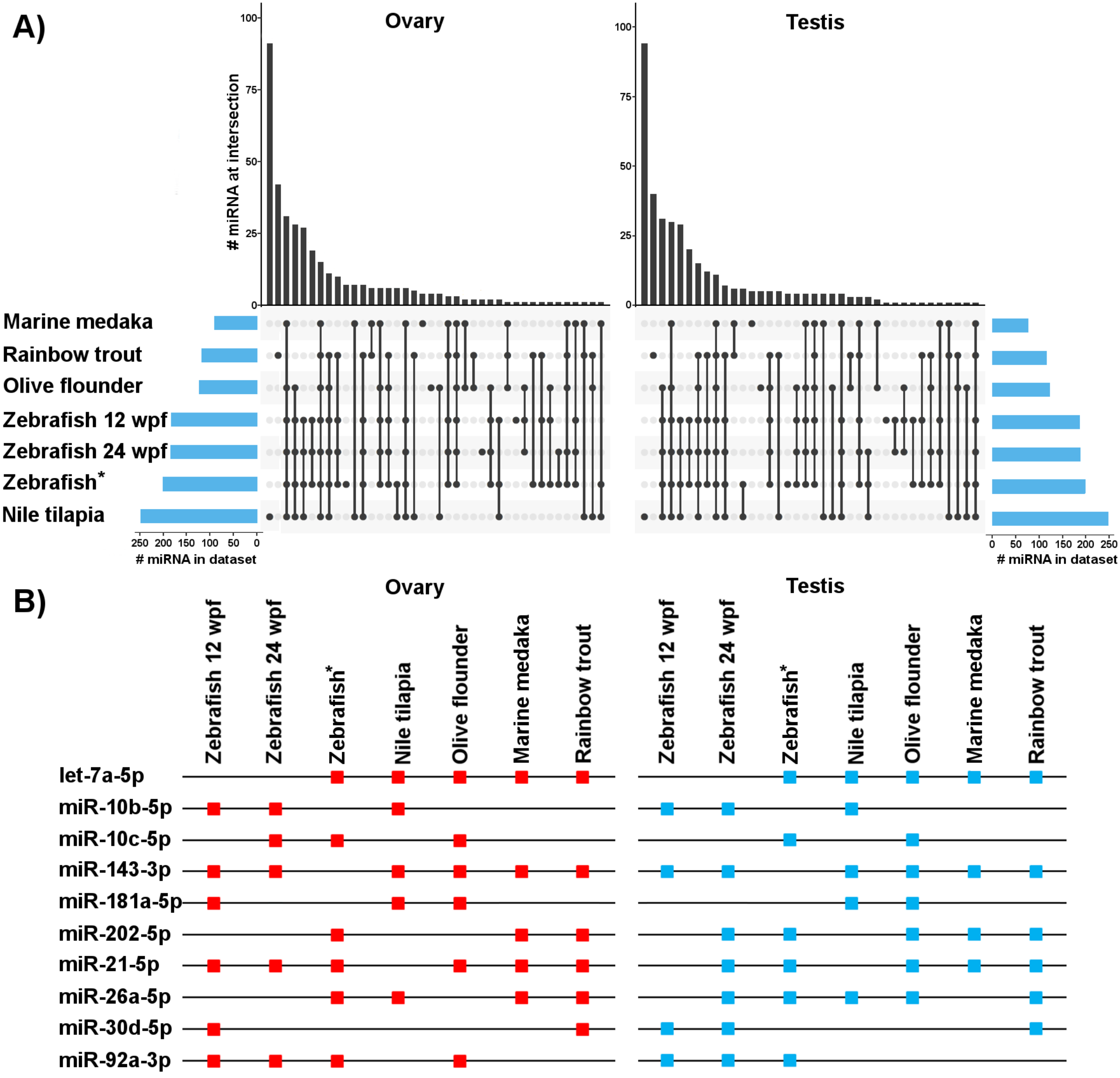

*Zebrafish dataset from Vaz et al. (2015)
